# Sertraline enhances the activity of antimicrobial agents against pathogens of clinical relevance

**DOI:** 10.1186/s40709-015-0028-1

**Published:** 2015-04-16

**Authors:** Muhammad Ayaz, Fazal Subhan, Jawad Ahmed, Arif-ullah Khan, Farhat Ullah, Ihsan Ullah, Gowhar Ali, Nawazish-i-Husain Syed, Sajid Hussain

**Affiliations:** Department of Pharmacy, University of Malakand, Khyber Pakhtoonkhwa, 18000 Pakistan; Department of Pharmacy, University of Peshawar, Peshawar, Pakistan; Institute of Basic Medical Sciences (IBMS), Khyber Medical University, Peshawar, Pakistan; Department of Pharmacology, Riphah Institute of Pharmaceutical Sciences, Riphah International University, Islamabad, Pakistan; Department of Pharmacy, University of Swabi, Swabi, Pakistan; University College of Pharmacy, University of The Punjab Lahore, Lahore, 54000 Pakistan; Department of Pharmacy, Kohat University of Science and Technology, Kohat, Pakistan

**Keywords:** Sertraline, Well assay, Minimum fungicidal concentration, ATCC

## Abstract

**Background:**

Serotonin reuptake inhibitors were recently reported to possess antimicrobial potentials, potentiate activity of several antibiotics, reverse multidrug resistant phenotypes of bacteria and make them susceptible to previously resistant drugs. We investigated antimicrobial potentials of sertraline (SR) against ATCC strains, clinical isolates of *Staphylococcus aureus*, *Escherichia coli* and *Pseudomonas aeruginosa* alone and in-combination with seven antibiotics. Antifungal activity was investigated against four fungal strains including *Aspergillus niger*, *Aspergillus fumigatus*, *Aspergillus flavus*, and *Fusarium solani.* Intrinsic antibacterial action and Minimum Inhibitory Concentrations (MICs) were determined using well assay, nutrient broth and agar dilution techniques. Disk diffusion and nutrient broth methods were used to study bacterial susceptibility to SR. Minimum Fungicidal Concentrations (MFCs) of SR were determined using Sabouraud dextrose Agar (SDA).

**Results:**

Sertraline possesses strong intrinsic antibacterial, antifungal activities and has augmented the antibacterial activities of antibiotics. For *S. aureus* ATCC 6538, *E. coli* ATCC 8739 and *P. aeruginosa* ATCC 9027, the MICs of SR were 20, 40 and 60 μg ml^−1^, respectively, whereas 55.5% clinical isolates of *S. aureus* and 50% of *E. coli* strains were inhibited at 20 and 60 μg ml^−1^ of SR, respectively. Among the tested fungi, 60% of *A. niger* and *A. fumigatus* were inhibited at 40 and 80 μg ml^−1^, respectively. MFCs were 60 and 80 μg ml^−1^ for *A. flavus* and *F. solani,* respectively*.* Antibacterial activities of all antibiotics were significantly increased (*p* < 0.001) with the addition of SR 100 μg ml^−1^ against all tested bacteria.

**Conclusion:**

Combination study revealed that SR had significantly increased the activity of antibiotics, and some previously resistant strains were made susceptible. Thus antidepressants are potential sources of resistance modifying agents when used in combination.

## Background

The origin of chemo-resistance is multifactorial and is primarily based on imprudent utilization, sustained over reliance on antimicrobial agents, target site modification and active drug efflux mediated by efflux pumps. To triumph over multidrug resistance (MDR), one strategy is the use of inhibitors of resistance mechanisms having the ability to augment the efficacy of existing chemotherapeutic agents. Based on this idea, antimicrobial drugs are co-administered with an efflux pump inhibitor (EPI) that will neutralize the acquired resistance and the drug will be still effective even against resistant microorganisms [1]. Other strategies include the use of antibiotic inactivating enzymes inhibitors, i.e. amoxicillin plus clavulanic acid [2] and inhibitors of efflux pumps [3,4].

A large number of compounds used in the management of non-infectious pathological conditions like inflammation, depression and cardiovascular diseases are known to exhibit antimicrobial activities and called non-antibiotics [5]. Various studies on the antimicrobial activities of non-antibiotic compounds including antidepressants [6,7], antipsychotic [8,9], antihypertensive [10], antihistaminics [11], antispasmodics [12], anti-inflammatory [13] and cardiovascular drugs [14] have been reported. In the presence of phenothiazines, including chlorpromazine and thioridazine in a culture medium, bacteria previously resistant to antimicrobial agents were shown to become susceptible [8,15]. More recently, it was suggested that chlorpromazine has decreased ethidium bromide efflux against all species of *Salmonella enterica, Mycobacterium avium* and *Mycobacterium smegmatis* [16,17]. Verapamil, a calcium channel blocker, has been reported to inhibit several bacterial efflux pumps including *p*-glycoprotein [18,19]. An anti-inflammatory drug diclofenac sodium was reported to possess remarkable antimicrobial properties against clinical isolates of bacteria [20]. Antidepressant drugs including selective serotonin reuptake inhibitors (SSRIs) have been extensively studied for their antimicrobial properties by several groups of researchers [6,21]. Kaatz *et al.* (2003) ivestigated antibacterial activities of paroxetine, femoxetine and their derivatives against *E. coli, S. aureus* and concluded that these compounds inhibit the activity of NorA, non-NorA and resistance nodulation division (RND) efflux pumps [22]. Similarly the antimicrobial properties of two tricyclic antidepressants imipramine, amitriptyline and phenothiazines were investigated by Hendricks *et al.* (2003) against *Klebsiella pneumoniae*, *Staphylococcus aureus* and *Pseudomonas aeruginosa* reported that these compounds exhibit excellent inhibitory effects against these bacteria [23]. Antidepressant drugs including fluoxetine, paroxetine, citalopram, and reboxetine have also been investigated by Lass-Flörl *et al.* for their antifungal properties against *Candida parapsilosis, Aspergillus fumigatus, Aspergillus terreus, Aspergillus flavus* and concluded these compounds inhibit the growth of all selected fungal strains and also posses inhibitory effect on replication of HIV [24,25].

Based on these evidences some antidepressants including sertraline (SR), citalopram (CIT) and venlafaxine (VF) were selected to uncover their potential antimicrobial characteristics alone, in-combination with antibiotics and effect of antidepressants-antibiotic combinations on resistance to these antibiotics. These antidepressants were also screened for antifungal potential against four pathogenic fungal strains. Among these drugs, SR was most potent regarding its minimum inhibitory concentration and intrinsic antibacterial activity and was further studied in combination with antibiotics.

## Results

### Intrinsic antibacterial assay of sertraline

**Figure 1:**
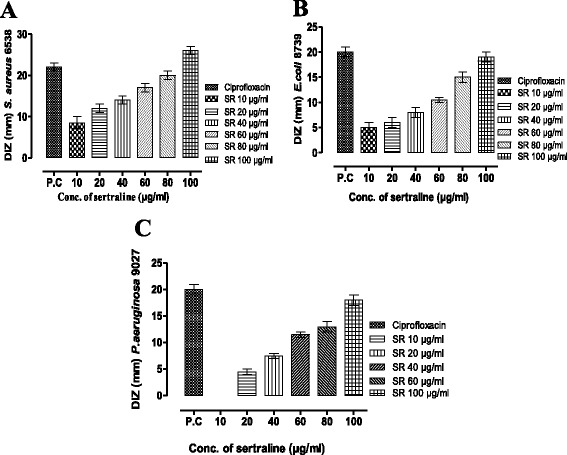
Intrinsic antibacterial effect of sertraline against *S. aureus* ATCC 6538 **(A)**, *E. coli* ATCC 8739 **(B)** and *P. aeruginosa* ATCC 9027 **(C)** *,* measured as diameter of inhibitory zone (DIZ) in comparison to positive control (Ciprofloxacin). DIZ (Diameter of inhibitory zone).

Using agar dilution and well assay methods for determination of intrinsic antibacterial activity of SR against *S. aureus,* DIZ were increased with the increasing concentrations of SR. DIZ were found to be 8.5, 12, 14, 17, 20 and 26 mm for 10, 20, 40, 60, 80 and 100 μg ml^−1^ of SR, respectively (Figure 1A). SR was found to possess strong antibacterial activity against *E. coli* 8739. SR in concentrations of 10, 20, 40, 60, 80 and 100 μg ml^−1^ scored mean inhibitory zones (n = 3) of 5, 6, 8, 11.5, 15 and 19 mm diameter, respectively against *E. coli* 8739 (Figure 1B). Similarly, the mean diameter of inhibitory zones of SR against *P. aeruginosa* was found to be 0, 4.5, 7.5, 11, 13 and 18 mm, respectively, against the same concentration of SR (Figure 1C).

### Minimum antibacterial and antifungal concentrations

MICs of SR against 28 bacterial strains including 3 ATCC, 25 clinical isolates and 13 fungal strains were studied. For ATCC strains (*S. aureus* ATCC 6538, *E. coli* ATCC 8739 and *P. aeruginosa* ATCC 9027), MICs of SR were 20, 40 and 60 μg ml^−1^, respectively. Regarding the clinical isolates including *S. aureus*, 22% (2/9) were inhibited at 10 μg ml^−1^, 55.5% (5/9) were inhibited at 20 μg ml^−1^ and 11% (1/9) were inhibited at 40 and 60 μg ml^−1^ of SR, respectively. In clinical isolates of *E. coli*, 18.75% (3/16) strains were inhibited at 10 μg ml^−1^, 12.5% (2/16) were inhibited at 20 and 40 μg ml^−1^, 50% (8/16) were inhibited at 60 μg ml^−1^ and 6.25% (1/16) were inhibited at 100 μg ml^−1^ of SR. For fungal strains, MFCs were 20 (1/5), 40 (3/5) and 80 (1/5) μg ml^−1^ for *A. niger,* 80 μg ml^−1^ (3/5) and 100 μg ml^−1^ (2/5) for *A. fumigatus,* 60 and 80 μg ml^−1^ for *A. flavus* and 80 μg ml^−1^ for *F. solani.*

### Effect of increasing concentrations of sertraline on the susceptibility of *S. aureus* 6538

Antibacterial studies of SR alone and in combination with antibiotics against S*. aureus* 6538 are summarized in Table 1. The Dunnett’s multiple comparison test was applied for the comparison of positive control with the test groups and revealed that a significant increase in the susceptibility pattern of *S. aureus* 6538 was observed. Diameter of Inhibitory Zone (DIZ) for ciprofloxacin in the absence of SR was 21.50 ± 0.70 mm, which was increased with the addition of SR in a concentration dependent manner and with the addition of SR 60 μg ml^−1^ a significant increase (*p* < 0.05) in the inhibitory zone (30 ± 4.24 mm) was observed. Likewise levofloxacin, norfloxacin and moxifloxacin exhibited significant synergy (*p* < 0.05) with SR at a concentration of SR 80 μg ml^−1^ scoring inhibitory zones of 33 ± 5.65 mm, 34.50 ± 7.77 mm, 34.50 ± 4.94 mm, respectively. The antibacterial activity of gentamicin was significantly increased at relatively low concentration of SR, i.e. 40 μg ml^−1^. *Staphylococcus aureus* 6538 resistance to cefixime and cloxacillin were not reversed at any concentration of SR being used.

**Table 1:** **Antibacterial effect of antibiotics alone and in combination with sertraline (SR) against** ***S. aureus*** **6538**

**Diameter of the inhibitory zone (mm) Mean ± SEM (n = 5)**							
**Antibiotic**	**Control**	**Antibiotic + SR 10 μ** **g ml** ^**−1**^	**Antibiotic + SR 20 μg ml** ^**−1**^	**Antibiotic + SR 40 μg ml** ^**−1**^	**Antibiotic + SR 60 μg ml** ^**−1**^	**Antibiotic + SR 80 μg ml** ^**−1**^	**Antibiotic + SR 100 μg ml** ^**−1**^
Ciprofloxacin 5 μg	21.50 ± 0.70	22.25 ± 0.50	24 ± 1.41	29 ± 1.40	30 ± 4.24*	37 ± 2.82**	39.5 ± 2.12**
Levofloxacin 5 μg	20.50 ± 0.70	21.70 ± 0.00	23 ± 1.41	25.50 ± 0.70	26.50 ± 0.70	33 ± 5.65*	36.5 ± 4.94**
Norfloxacin 10 μg	16.50 ± 0.70	17.5 ± 0.50	20.50 ± 2.12	21 ± 1.41	24 ± 1.41	34.5 ± 7.77*	36.5 ± 7.77*
Moxifloxacin 5 μg	22 ± 1.41	22 ± 0.00	23 ± 1.41	26.50 ± 0.70	28.50 ± 0.70	34.50 ± 4.94*	37.5 ± 3.53**
Cefexime 5 μg	0 ± 0.00	0 ± 0.00	0 ± 0.00	0 ± 0.00	0 ± 0.00	0 ± 0.00	0 ± 0.00
Cloxacillin 5 μg	0 ± 0.00	0 ± 0.00	0 ± 0.00	0 ± 0.00	0 ± 0.00	0 ± 0.00	0 ± 0.00
Gentamicin10 μg	21.50 ± 0.70	22.50 ± 0.20	24.5 ± 0.70	27.50 ± 0.70**	29 ± 1.41**	35 ± 1.41***	36.5 ± 0.7***

Significantly different as compared to antibiotic treated group only: *p < 0.05, **p < 0.01, ***p < 0.001. Each value represent the mean of five replicates (n = 5). Control: Antibiotic treated group only.

### Effect of increasing concentrations of sertraline on the susceptibility of *E. coli* 8739

The DIZ for ciprofloxacin in the absence of SR was 20 ± 1.41 mm which was increased significantly (23 ± 1.41 mm, *p* < 0.05) with the addition of 20 μg ml^−1^ of SR (Table 2). Susceptibility to antibiotics increases with the addition of SR in concentration dependent manner. Levofloxacin, norfloxacin, and gentamicin exhibited significant synergy (*p* < 0.05) with SR at 40 μg ml^−1^ scoring inhibitory zones of 22 ± 0.50 mm, 25.50 ± 0.70 mm, and 29.5 ± 0.70 mm, respectively. Antibacterial effect of moxifloxacin against *E. coli* 8739 was significantly increased (*p* < 0.001) at 80 μg ml^−1^ with an inhibitory zone of 32 ± 1.41 mm. *Escherichia coli* 8739 was initially resistant to cefixime but at concentration of 80 μg ml^−1^ it was made susceptible with an inhibitory zone of 9.50 ± 0.70 mm. At still higher concentrations of SR susceptibility was further increased scoring an inhibitory zone of 13.50 ± 2.12 mm. *Escherichia coli* 8739 was resistant to cloxacillin and the resistance was not affected by increasing concentrations of SR.

**Table 2:** **Antibacterial effect of antibiotics alone and in combination with increasing concentrations of sertraline (SR) against** ***E. coli*** **87**

**Antibiotic**	**Diameter of the inhibitory zone (mm) Mean ± SEM (n = 5)**						
	**Control**	**Antibiotic + SR 10 μg ml** ^**-1**^	**Antibiotic + SR 20 μg ml** ^**-1**^	**Antibiotic + SR 40 μg ml** ^**-1**^	**Antibiotic + SR 60 μg ml** ^**-1**^	**Antibiotic + SR 80 μg ml** ^**-1**^	**Antibiotic + SR 100 μg ml** ^**-1**^
Ciprofloxacin 5 μg	20 ± 1.41	21 ± 00	23 ± 1.41*	23.5 ± 0.70**	30 ± 0.00***	33.5 ± 2.10***	39 ± 1.41***
Levofloxacin 5 μg	18 ± 1.41	20 ± 0.50	21 ± 0.00	22 ± 0.50*	25 ± 0.00**	29 ± 0.00***	38 ± 1.41***
Norfloxacin 10 μg	17 ± 0.41	17 ± 0.00	19.50 ± 2.12	25.50 ± 0.70*	24.50 ± 0.70**	27.50 ± 0.70***	35.50 ±0.70***
Moxifloxacin 5 μg	20.5 ± 0.70	21 ± 0.12	22.50 ± 0.70	27.50 ± 0.70	27.50 ±0.70	32 ± 1.41***	40.50 ± 0.70***
Cefexime 5 μg	0 ± 0.00	0 ± 0.00	0 ± 0.00	0 ± 0.00	0 ± 0.00	9.50 ± 0.70	13.50 ± 2.12
Cloxacillin 5 μg	0 ± 0.00	0 ± 0.00	0 ± 0.00	0 ± 0.00	0 ± 0.00	0 ± 0.00	0 ± 0.00
Gentamicin 10 μg	23 ± 2.82	24 ± 0.70	25 ± 2.82	29.5 ± 0.70*	31.50 ± 0.70**	34.50 ± 0.70**	41.50 ± 0.70***

Values significantly different as compared to antibiotic treated group only: *p < 0.05, **p < 0.01, ***p < 0.001. Each value represent mean of five replicates (n = 5). Control: Antibiotic treated group only.

### Effect of increasing concentrations of sertraline on the susceptibility of *P. aeruginosa* 9027

Antibacterial effect of antibiotics was increased significantly against *P. aeruginosa* 9027 with the addition of increasing concentrations of SR. Results are summarized in Table 3. DIZ for all antibiotics increased with the addition of SR in a concentration dependent manner. DIZ for levofloxacin (22 ± 1.41 mm), norfloxacin (20 ± 0.00 mm) and moxifloxacin (24 ± 1.41 mm) were significantly increased with the addition of SR 40 μg ml^−1^, SR 20 μg ml^−1^ and SR 60 μg ml^−1^ of SR respectively. *Pseudomonas aeruginosa* 9027 was completely resistant to cefixime and cloxacillin and addition of increasing concentrations of SR has not reversed this resistance. In the absence of SR the DIZ for gentamicin against *P. aeruginosa* 9027 was 24.50 ± 0.70 mm which was increased significantly (*p* < 0.05) at concentration of SR 40 μg ml^−1^ of SR scoring an inhibitory zone of 29.50 ± 0.70 mm.

**Table 3:** **Antibacterial effect of sertraline (SR) and antibiotics combination against** ***P. aeruginosa*** **9027**

**Diameter of the inhibitory zone (mm) Mean ± SEM (n = 5)**							
**Antibiotic**	**Control**	**Antibiotic + SR 10** **μg ml** ^**−1**^	**Antibiotic + SR 20 μg ml** ^**−1**^	**Antibiotic + SR 40 μg ml** ^**−1**^	**Antibiotic + SR 60 μg ml** ^**−1**^	**Antibiotic + SR 80 μg ml** ^**−1**^	**Antibiotic + SR 100 μg ml** ^**−1**^
Ciprofloxacin 5 μg	20 ± 0.00	20.8 ± 0.20	22 ± 2.82	26 ± 2.82	29 ± 2.82*	33 ± 2.82**	40 ± 2.82***
Levofloxacin 5 μg	22 ± 1.41	23.0 ± 0.50	24 ± 1.41	27 ± 1.41*	30 ± 1.41**	37 ± 1.41***	41 ± 0.00***
Norfloxacin 10 μg	20 ± 0.00	21 ± 0.70	24.5 ± 0.70**	26.50 ± 0.70***	30.50 ± 0.70***	33.5 ± 0.70***	39.5 ± 0.70***
Moxifloxacin 5 μg	24 ± 1.41	24 ± 0.50	26 ± 1.41	28 ± 1.41	32 ± 1.41**	36 ± 1.41***	42.5 ± 0.70***
Cefexime 5 μg	0 ± 0.00	0 ± 0.00	0 ± 0.00	0 ± 0.00	0 ± 0.00	0 ± 0.00	0 ± 0.00
Cloxacillin 5 μg	0 ± 0.00	0 ± 0.00	0 ± 0.00	0 ± 0.00	0 ± 0.00	0 ± 0.00	0 ± 0.00
Gentamicin 10 μg	24.50 ± 70	25 ± 0.50	26.50 ± 0.70	29.50 ± 0.70*	33.50 ± 0.70***	38.50 ± 0.70***	41.5 ± 0.70***

Values significantly different as compare to antibiotic treated group only: *p < 0.05, **p < 0.01, ***p < 0.001. Control: Antibiotic treated group only.

## Discussion

Combination therapy with antimicrobial agents has become commonplace due to the emergence of multidrug resistant (MDR) pathogens [26,27]. One of the strategies is to develop drugs in combination, as synergistic interactions may potentially prevent the emergence of acquired resistance, can augment efficacy, decrease toxicity and provide broader spectrum of activity than mono-therapy regimens. Previous studies demonstrated that the combination antimicrobial therapy is effective against difficult-to-treat diseases like tuberculosis and HIV infection, as these microbes do not respond to mono-therapy either due to emergence of resistance or lack of efficacy [28]. As combination drug therapy is a suitable model of additively, so such experiments can provide important insights into the significance of synergistic and antagonistic relations of a variety of compounds and antimicrobials [29]. In this regard, antibiotics and non-antibiotic combinations should be checked for possible synergistic or antagonistic interactions. Identification of such combinations might be beneficial for empirical use and can decrease the cost and duration of antimicrobial drug therapy. The motive for selecting SR for synergy studies was long term use and previous studies on antidepressants and antipsychotic drugs [22,23,25]. Our findings indicate that SR possess strong antibacterial and antifungal activities *in vitro* and some bacterial strains previously resistant to antibiotics were made susceptible with the addition of SR.

From the intrinsic antibacterial studies, it is evident that SR possesses antibacterial characteristics and inhibited bacterial growth at different concentrations. The antibacterial activity of SR was more prominent against gram positive bacteria, i.e. *S. aureus* as compared to gram negative. Diameter of inhibitory zones were increased significantly along the concentration of 10, 20, 40, 60, 80, 100 μg ml^−1^ and at still higher concentrations complete inhibition of bacterial growth was observed. Using nutrient broth, MIC study revealed that the most effective concentration of SR against *S. aureus* was 20 μg ml^−1^ at which majority of clinical isolates and ATCC strain were inhibited while 60 μg ml^−1^ was most effective against *E.coli* strains indicating its effectiveness against Gram positive bacteria. Antifungal study demonstrates that SR possess fungicidal properties and inhibited fungal growth at different concentrations. SR in a concentration of 80 μg ml^−1^ was most effective, at which majority of fungal strains were inhibited. Interestingly, majority of *A. niger* species were inhibited at relatively low concentration of SR, i.e. 40 μg ml^−1^. Previous studies indicated that chlorpromazine and its analogues possesses bactericidal, fungicidal, and antiprotozoal properties through enzymatic inhibition but the proposed bactericidal and fungicidal activities of SR is still to be uncovered [30]. However it has been reported that the antifungal activity of SR is probably due to interaction with the fungal membrane transport system [24] or through inhibition of extracellular phospholipase activity [25] leading to cell death.

During combination studies, synergy between antibiotics and non-antibiotic (SR) was observed. Antibiotics exhibited synergy with SR against *S. aureus* in a concentration dependent manner, and diameter of inhibitory zones were increased with the addition of increasing concentrations of SR. However *Staphylococcus* species showed resistance to cefixime and cloxacillin and with the addition of SR susceptibility to these antibiotics were not changed. Addition of SR at concentrations higher than 100 μg ml^−1^ with antibiotic has completely eradicated bacterial growth and as a result quantification of antibiotic-SR synergy at this concentration was not possible. The exact mechanism of antibacterial activity of SR is still not clear and will require molecular studies. We hypothesize that as SR is selective serotonin reuptake inhibitor (SSRI), a reuptake pump inhibitor in humans [31], so it can act as efflux pump inhibitor in bacteria. However, to confirm that antibacterial activity of SR is due to inhibition of efflux pump, use of bacteria with molecularly characterized efflux pumps and studies with known efflux pumps inhibitors like ethidium bromide will be required. In this regard further studies are in progress in our laboratory.

The inhibitory zones were increased in the same way for the selected antibiotics with the addition of increasing concentrations of SR against *E. coli.* Initially resistance to cefixime was observed however with the addition of increasing concentrations of SR *E. coli* species were converted to susceptible range. Increase in the antibacterial effect of antibiotics was sustained in the same manner for *P. aeruginosa.* However its resistance to cefixime and cloxacillin were not affected by addition of SR.

The human therapeutic dose of SR is 50–200 mg daily [32], so the concentrations being used are very low as compared to minimum toxic concentrations and safety studies at high concentrations, i.e. 1300 μg ml^−1^ in experimental animals are already established [33]. From bioavailability point of view any relevance between our *in vitro* results and *in vivo* performance is still not clear and may require further *in vivo* studies to investigate the mechanism of SR induced bacterial and fungal death. However it is concluded that SR and other SSRIs are potential drugs for further characterization and development of new antimicrobial drugs. Further, while using SR for antimicrobial purpose, its systemic effects including neurological aspects especially at high doses must be determined. Further studies are required in this regard.

## Conclusions

Sertraline (SR) possesses strong intrinsic antibacterial and antifungal activities. Combination study revealed that sertraline has significantly increased the antimicrobial effect of antibiotics, and some previously resistant strains were made susceptible. Further, SR is very effective at higher concentration but neurological effects at such higher concentration must be studied. Further derivatization and use of bacteria containing molecularly characterized efflux pumps can provide more convincing results.

## Methods

### Chemicals and Drugs

Sertraline (SR) was kindly provided by Wilshire Pharmaceuticals Lahore, Pakistan. Antibiotic powder of ciprofloxacin, levofloxacin, norfloxacin, moxifloxacin, cefixime, cloxacillin*,* and gentamicin*,* (Sigma Aldrich CHEMIE GmbH USA) were used in the study. Dimethyl-Sulfoxide (DMSO) (Labscan Patumwan Bankok 10330 Thialand), 99.9% pure was used for dissolution of SR.

### Bacteria and fungi

Gram positive, Gram negative and American Type Culture Collection (ATCC) strains of bacteria and fungal strains were used to uncover the antibacterial and antifungal potential of SR. Nine clinical isolates and one ATCC (6538) strain of *S. aureus*, 16 clinical isolates and one ATCC strain (8739) of *E. coli*, one ATCC strain of *P. aeruginosa* (9027), 13 fungal strains of *A, niger*, *A. fumigatus*, *A. flavus*, and *F. solani* were used in the study. Bacterial ATCC strains were provided by Cirin Pharmaceuticals Pakistan. Clinical isolates were collected from microbiology department of Khyber Teaching Hospital (KTH) Peshawar, Pakistan and were identified by different biochemical tests [34]. Bacteria were preserved in freeze-dried condition at 4°C in stab slant agar until later use. Fungal strains were provided by Microbiology Department Kohat University of Science and Technology (KUST) Kohat, Pakistan.

### Culture media

Mueller-Hinton agar (MHA Oxoid UK), Mannitol salt agar, Mackonkey’s Agar (Oxoid Ltd, England, CM0115), Triple sugar iron (TSI) agar (Oxoid Ltd, England, CM0277), Sabouraud’s dextrose agar and nutrient broth base powder (Oxoid Ltd, England) were used in the study for culture and identification of microbes according to the guidelines of clinical laboratory standards institute (CLSI) and manufacturer specifications [35].

### Preparation of sertraline stock solutions

SR is sparingly soluble in water so it was dissolved in DMSO and serial two fold dilutions of the drug were made, ranging from 10–100 μg ml^−1^ under laminar flow hood. Stock solutions and dilutions of the drugs were prepared according to CLSI recommendations and manufacturer’s specifications [36,37].

### Standardization of bacterial and fungal suspensions

Bacterial culture was grown for 24 hrs at 37°C and suspension with cell density of 1 × 10^8^ CFU ml^−1^, was prepared using McFarland standard and was further diluted to a cell density of 1 × 10^6^ CFU ml^−1^ using a UV visible spectrophotometer (Thermo electron corporation, USA) at 625 nm and the standardization was maintained for the period of the study. Fungal strains were grown at 25°C and suspensions corresponding to 2.5 × 10^4^ cells ml^−1^ were prepared by dilution in normal saline. Standardization of fungal strains were performed using microscopic enumeration with a cell-counting hematocytometer and optical density method as previously reported [38].

### Intrinsic antibacterial studies on sertraline

Preliminary intrinsic antibacterial action of SR was determined using agar dilution and well assay methods. In agar dilution method, SR solutions corresponding to 10, 20, 40, 50 60, 80 and 100 μg ml^−1^ were aseptically added to sterile molten MHA at 40°C [39]. One loopful of the already prepared bacterial suspension was inoculated on the MHA plates containing increasing concentration of SR. Plates were incubated at 37°C for 18–72 hrs and were examined for the appearance of growth. In well assay method, bacterial plates were inoculated by swabbing MHA plates with already prepared bacterial suspensions under laminar flow hood [40-42]. Wells of 6 mm diameter were bored into the MHA plates using sterilized cork borer. After drying the bores were filled with 100 μL solutions of different concentrations of SR and antibiotics taking care not to let spillage of the solutions on the surface of the agar. The plates were incubated at 37°C for 24 hrs. Zone of inhibition were measured around the bores and were compared with positive control ciprofloxacin.

### Determination of minimum inhibitory concentration (MICs)

For determination of MICs both nutrient broth and agar dilution methods approved by national committee for clinical laboratory standards (NCCLS) were used [43,44]. For these tests, SR in concentration of 10, 20, 40, 50 60, 80 and 100 μg ml^−1^ were added to sterilized tube containing nutrient broth and were inoculated with the test microbes. Tubes were incubated using shaker incubator at 37°C for 24 hrs. MIC was considered that concentration at which no visible bacterial growth was observed. All experiments were done in three replicates.

### *In vitro* synergy between sertraline (SR) and antibiotics

The combined antibacterial effect of SR and antibiotics was determined by disk diffusion method described by CLSI [45] and previously reported procedure by Dutta *et al*. [20]. Sterile filter paper disks (Whatman no. 1) 7.25 mm diameter were prepared. In combination study of SR and antibiotics, solutions corresponding to 5 μg of ciprofloxacin, levofloxacin, moxifloxacin, cefexime, cloxacillin, 10 μg of norfloxacin and gentamicin and 10–100 μg of SR were added to these disks. Antibiotic disks of these drugs available were used as positive control. Overnight grown bacterial culture was used to prepare bacterial suspensions. From these suspension 100 μl were uniformly spread over the surface of already prepared MHA plates under laminar flow hood and were allowed to dry. Disks containing antibiotic alone and increasing concentrations of SR were placed equidistantly on the surface of inoculated plated and were incubated at 37°C for 24 hrs. Diameter of inhibitory zones (DIZ) of antibiotic alone and in combination with SR was determined to CLSI standards for zone interpretation [46].

### Antifungal activities of sertraline

Minimum fungicidal concentrations (MFC) of SR were determined using Sabouraud’s dextrose agar (SDA). Briefly, Sabouraud’s dextrose agar tubes were prepared according to manufacturer specifications and solutions of SR corresponding to 10, 20, 40, 50 60, 80 and 100 μg ml^−1^ were added to these tubes at 40°C. The tubes were inoculated by adding one loopful of already prepared fungal suspensions and were incubated at 27°C. After 7–10 days of incubation tubes were observed for fungal growth. MFC were considered the lowest concentration that inhibited fungal growth nn.

### Statistical analysis

One-way ANOVA, followed Dunnett's multiple comparison test, were applied for the comparison of positive control with the test groups using GraphPad Prisim-5 (Software Inc., La Jolla, CA, USA). All the assays were repeated in triplicate and values were expressed as means ± S*.*E*.*M. The *p* values less than 0.05 were considered as statistically significant.
